# Management of Hepatocellular Carcinoma in the Era of Individualized Therapy: The Experience of a Greek Tertiary Center

**DOI:** 10.31486/toj.19.0092

**Published:** 2020

**Authors:** Chrysanthos D. Christou, Andreas Tooulias, Alexandros Tsolakidis, Vassilis Papayiannis, Bozidaria Pianetcki-Tsiantzi, Georgios Tsoulfas, Vasileios N. Papadopoulos

**Affiliations:** ^1^ Department of First General Surgery, Papageorgiou General Hospital, Thessaloniki, Greece; ^2^ Department of Radiology, Papageorgiou General Hospital, Thessaloniki, Greece; ^3^ Department of Anesthesiology, Papageorgiou General Hospital, Thessaloniki, Greece; ^4^ Department of First General Surgery, Aristotle University of Thessaloniki, Thessaloniki, Greece

**Keywords:** *Carcinoma–hepatocellular*, *catheter ablation*, *chemoembolization–therapeutic*, *liver neoplasms*, *neoplasm staging*

## Abstract

**Background:** The treatment of hepatocellular carcinoma (HCC) in the era of individualized therapy mandates a multidisciplinary approach and therefore the cooperation of physicians from multiple medical specialties. Treatment selection is based on the stage of the disease. The most prominent staging system is the Barcelona Clinic Liver Cancer (BCLC) classification system.

**Methods:** We conducted a retrospective cohort study of patients with HCC treated in our department. Patients were originally staged based on the BCLC classification system. However, a multidisciplinary team refined the BCLC classes, using clinical data and biomarkers to tailor an individualized course of treatment.

**Results:** The study population was 63 patients who were BCLC staged at diagnosis as follows: very early (5 patients, 7.9%), early (38 patients, 60.3%), intermediate (14 patients, 22.2%), and advanced (6 patients, 9.5%). Thirty-two patients (50.8%) were treated with surgery and 31 patients (49.2%) with locoregional treatments. The 1-year, 3-year, and 5-year survival rates in the surgery group were 81.3%, 52.9%, and 18.9%, respectively, whereas in the locoregional treatment group, the 1-year, 3-year, and 5-year survival rates were 71.0%, 38.7%, and 19.0%, respectively. The mean overall survival was 35.42 ± 23.54 months for the surgery group and 28.42 ± 23.0 months for the locoregional treatment group. In the surgery group, the mean overall survival of the patients treated with surgery alone was 26.68 ± 21.97 months compared to 48.18 ± 20.26 months for the patients treated with surgery followed by locoregional treatment for recurrence.

**Conclusion:** In this study, patients treated with hepatic resection had higher survival rates than patients treated with locoregional treatments. However, this superiority did not reach statistical significance (*P*=0.426). Thus, locoregional treatments are highlighted as a valuable alternative to surgery, particularly when hepatic resection is not feasible. Finally, patients who received locoregional treatment following surgery had significantly higher survival compared to patients treated with surgery alone (*P*=0.038).

## INTRODUCTION

Hepatocellular carcinoma (HCC) is the fifth most common malignancy and the third most common cause of death related to cancer worldwide.^1^ In Europe in 2018, 82,470 new patients were diagnosed with HCC, while 77,370 died from HCC.^2^ The treatment of HCC in the era of individualized therapy mandates a multidisciplinary approach that involves physicians from various medical specialties. The arrows in the quiver against HCC are plenty. The most prominent include liver transplantation; hepatic resection; ablation techniques such as radiofrequency ablation (RFA) and microwave ablation; transarterial chemoembolization (TACE); and systemic therapies such as sorafenib, lenvatinib, and regorafenib.^3,4^ The cornerstone of treatment selection is the stage of the disease.^5,6^ A plethora of staging systems has been proposed. In Europe and the United States, guidelines for the management of HCC have been developed based on the Barcelona Clinic Liver Cancer (BCLC) staging classification system.^3,7^

Regarding treatment, liver transplant and hepatic resection remain the gold standard for very early and early stage patients with HCC. For intermediate and advanced stage HCC, the guidelines recommend TACE and oral sorafenib treatment, respectively.^3,7^ For patients who progress on sorafenib, treatment with regorafenib and radioembolization has been associated with increased survival.^8,9^ For terminal stage patients with HCC, only supportive care is provided since there is no indication for tumor-directed treatments.^3^

Transplantation is often not an available treatment option in many countries, including Greece, principally because of the shortage of grafts. Based on Global Observatory on Donation and Transplantation data, the total liver transplants per million of population (both from living and deceased patients) in 2018 was 2.07 in Greece vs 25.24 in the United States.^10^ This extensive lack of grafts transforms an actual treatment option into a utopian dream. As a result, physicians in many countries are required to choose alternative courses of treatment to transplantation. Our study provides our experience in the multidisciplinary treatment of HCC using hepatic resection, TACE, and ablation techniques under the BCLC guidelines.

## METHODS

### Study Design and Patient Selection

This study was conducted at the Papageorgiou General Hospital (PGH) in Thessaloniki, the second most populous city in Greece. As a tertiary hospital, PGH receives patients from Thessaloniki and patients referred by secondary hospitals from the surrounding geographic regions, thus serving a region that represents approximately 15% of the country's population. Our study was conducted in accordance with the 1964 Declaration of Helsinki and its later amendments, and the hospital institutional board review (IRB) granted a waiver of patient consent because all data were extracted from the patients’ medical records in an anonymized manner with no risk of personal data identification. Following IRB approval, we performed a comprehensive search of our department's records to identify all patients who were treated for HCC (International Classification of Diseases-10 code C22.0) from March 1, 2010 through November 28, 2019.

### Preoperative Assessment and Interventions

As recommended by the European Association for the Study of the Liver (EASL),^6^ our surgical team assesses our patients upon diagnosis based on performance status, tumor burden, and liver function, and patients are originally staged based on the BCLC system. Subsequently, patients are discussed in an oncology board that consists of physicians of multiple specialities, including oncologists, internists, radiologists, anesthesiologists, and surgeons. This multidisciplinary team, using clinical data and biomarkers, refines the BCLC classes to tailor an individualized course of treatment for each patient. As a result, a patient may receive a treatment that does not correspond to his or her BCLC class. During follow-up, the oncology board assesses patients multiple times to evaluate the progression of their disease and determine further treatment.

Hepatectomies are performed by 4 general surgeons, 2 senior staff and 2 residents; 2 anesthesiologists, 1 senior staff and 1 resident; and 2 surgical nursing staff. All hepatic resections are radiofrequency-assisted regarding the transection of the liver. Vascular occlusion techniques are not performed. All hepatic resections are described using the 2000 Brisbane nomenclature.^11^ TACE is performed under local anesthesia through the right common femoral artery. Selective and superselective angiographic runs are performed to reveal all the vascular anatomy of the liver, as well as any existing variants or parasitic vasculature to the tumor. All feeding arteries to the tumor are then superselectively catheterized, and a homogeneous mixture of the indicated chemotherapeutic agent is injected.

### Data Collection and Definition of Outcomes

For each patient, we collected information regarding demographics, comorbidities, radiologic and laboratory characteristics of the disease, staging of the disease at the time of diagnosis, therapeutic procedures, and overall survival. We also collected information on the type of procedure (surgery, TACE, RFA), length of hospitalization, and perioperative and 30-day mortality. Length of hospitalization and 30-day mortality were calculated considering the day of the surgery as day 0. Survival was calculated in months from the day of confirmed diagnosis by either computed tomography scan or biopsy until the day of death. The exact date of death was obtained from National Healthcare System data. Patients who were still alive were censored from the study based on their last follow-up date.

### Statistical Analysis

The statistical analysis was performed using SPSS v.25.0 (IBM Corp.). Categorical variables are described using frequencies and percentages. Quantitative variables are presented as means ± SD. Survival statistics are presented using the Kaplan-Meier survival curves and survival tables, censoring patients who were still alive. Survival curves were compared using the Mantel-Cox test (log-rank test). Pairwise comparisons over strata were conducted to compare survival among the different BCLC stages. Any test with *P* value ≤0.05 was considered statistically significant.

## RESULTS

### Patient Demographics

The study population consisted of 63 patients with HCC. Baseline characteristics of patients at the time of diagnosis are presented in Table 1. The population included more males than females, with a ratio of approximately 4:1. The patients’ mean ages at diagnosis were comparable: 68.6 ± 1.44 years in the male group and 64.92 ± 2.62 years in the female group.

**Table 1.** Characteristics of Patients at Time of Diagnosis (n=63)

Variable	Value
Mean age, years ± SD	67.8 ± 1.27
Sex	
Male	50 (79.4)
Female	13 (20.6)
Comorbidities	
Alcohol consumption	20 (31.7)
Hepatitis B virus positive	26 (41.3)
Hepatitis C virus positive	4 (6.3)
Hypertension	31 (49.2)
Bilirubin level, mg/dL	
<2	54 (85.7)
2-3	5 (7.9)
>3	4 (6.3)
Albumin level, g/dL	
<2.8	4 (6.3)
2.8-3.5	12 (19.0)
>3.5	47 (74.6)
International normalized ratio <1.7	63 (100)
Alpha-fetoprotein level, ng/mL	
<200	51 (81.0)
200-500	4 (6.3)
>500	8 (12.7)
Ascites	
None	48 (76.2)
Controlled	15 (23.8)
Model for End-Stage Liver Disease score	
6-11	49 (77.8)
12-14	10 (15.9)
15-19	4 (6.3)

Note: All data are presented as n (%) unless otherwise noted.

### Staging of the Disease and Treatment Groups

**Figure 1.**
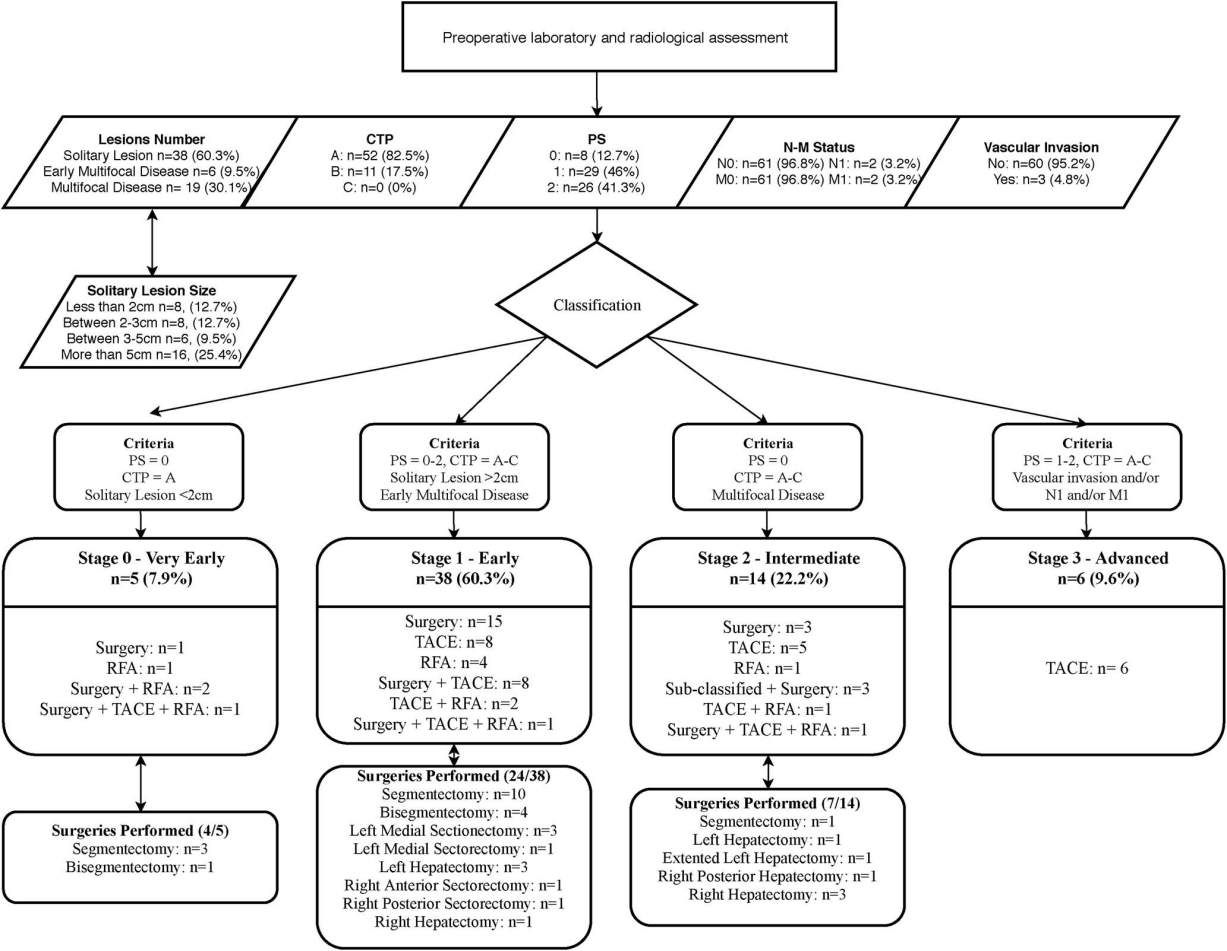
**Patient assessments, classifications, and treatments (n=63).** Hepatic resections are described using the Brisbane 2000 nomenclature. CTP, Child-Turcotte-Pugh Score; PS, performance status; RFA, radiofrequency ablation; TACE, transarterial chemoembolization.

Laboratory and radiologic characteristics were used to classify patients’ disease based on the Child-Turcotte-Pugh (CTP) and BCLC staging systems. Figure 1 illustrates the classification procedure and correlates the stage of the disease with the course of treatment.

Patients were divided into 2 treatment groups: the surgery group and the locoregional group. Thirty-two patients (50.8%) underwent a surgical intervention, and 31 patients (49.2%) received locoregional treatment.

In the surgery group (n=32), 13 patients (40.6%) also received locoregional treatment—TACE, RFA, or both—for recurrence. Nine patients (28.1%) were admitted to the intensive care unit following surgery. The mean length of hospitalization for the surgery group was 9.77 ± 3.53 days. Perioperative and 30-day mortality rates in the surgery group were 0% and 6.3%, respectively (n=2).

In the locoregional group (n=31), 19 patients (61.3%) were treated with TACE, 6 patients (19.4%) with RFA, and 3 patients (9.7%) with a combination of both techniques. The remaining 3 patients (9.7%) originally received locoregional treatment because their carcinomas were defined as unresectable, but during restaging following the locoregional interventions, their carcinomas were downsized and downstaged to resectable.

### Survival Statistics

The mean overall survival of our patients was 31.97 ± 23.36 months. Table 2 provides the survival statistics by disease stage and treatment group.

**Table 2.** Patient Survival by Disease Stage and Treatment Group

Stage/Treatment	Mean Overall Survival, months ± SD	1-Year Survival, %	3-Year Survival, %	5-Year Survival, %
Disease stage^a^				
Very early (n=5)	51.18 ± 14.70	100	100	50.0
Early (n=38)	35.14 ± 24.0	84.2	47.4	20.7
Intermediate (n=14)	27.76 ± 19.64	71.4	41.7	8.3
Advanced (n=6)	5.79 ± 3.15	0	0	0
Treatment group				
Surgery (n=32)	35.42 ± 23.54	81.3	52.9	18.9
Surgery alone (n=19)	26.68 ± 21.97	68.4	36.8	22.1
Surgery and locoregional (n=13)	48.18 ± 20.26	100	76.9	17.1
Locoregional (n=31)	28.42 ± 23.0	71.0	38.7	19.0

^a^Disease stage was determined by the Barcelona Clinic Liver Cancer staging classification system.

**Figure 2.**
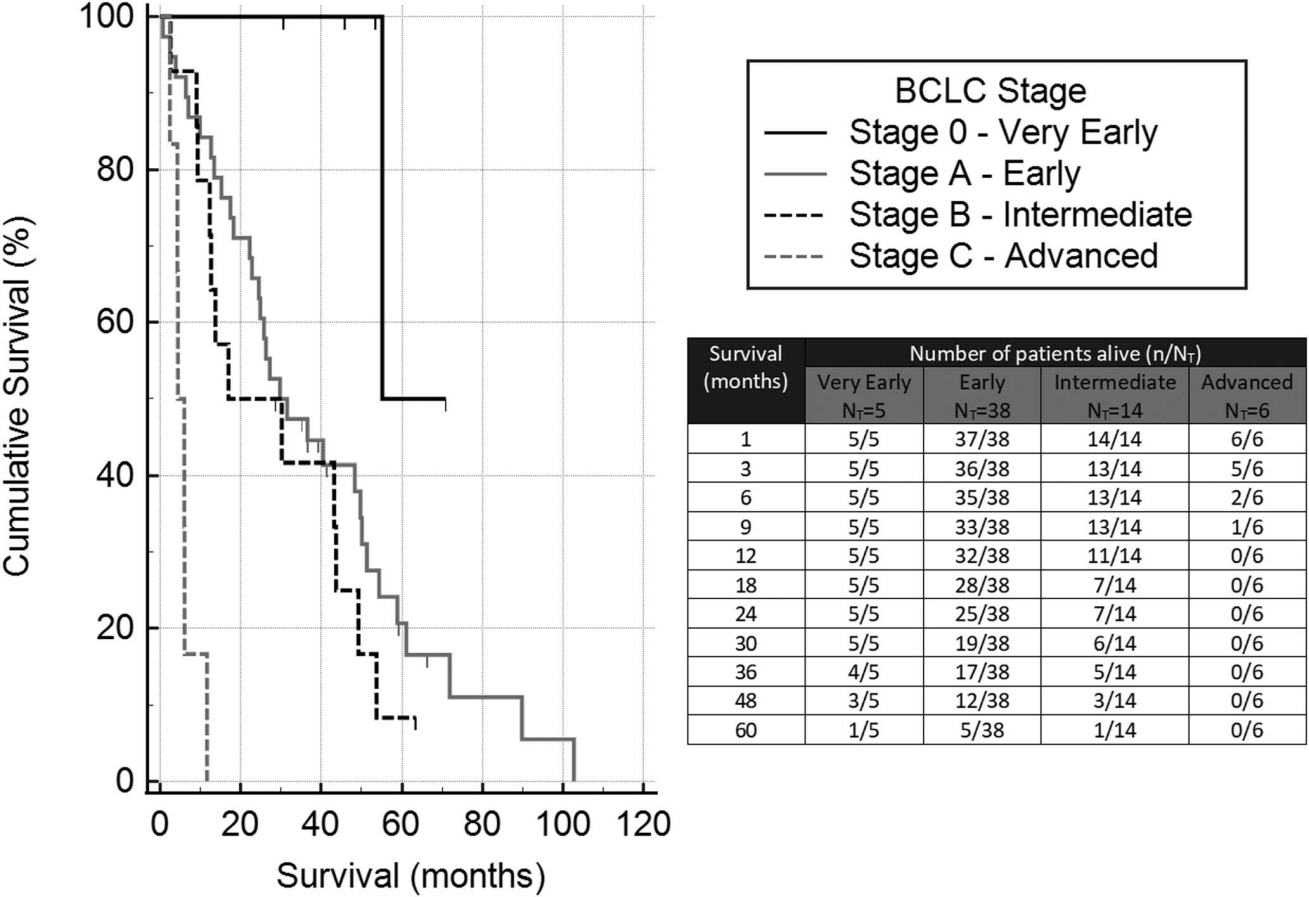
**Kaplan-Meier survival analysis of overall survival based on the stage of the disease.** N_T,_ total number of patients in the group.

**Figure 3.**
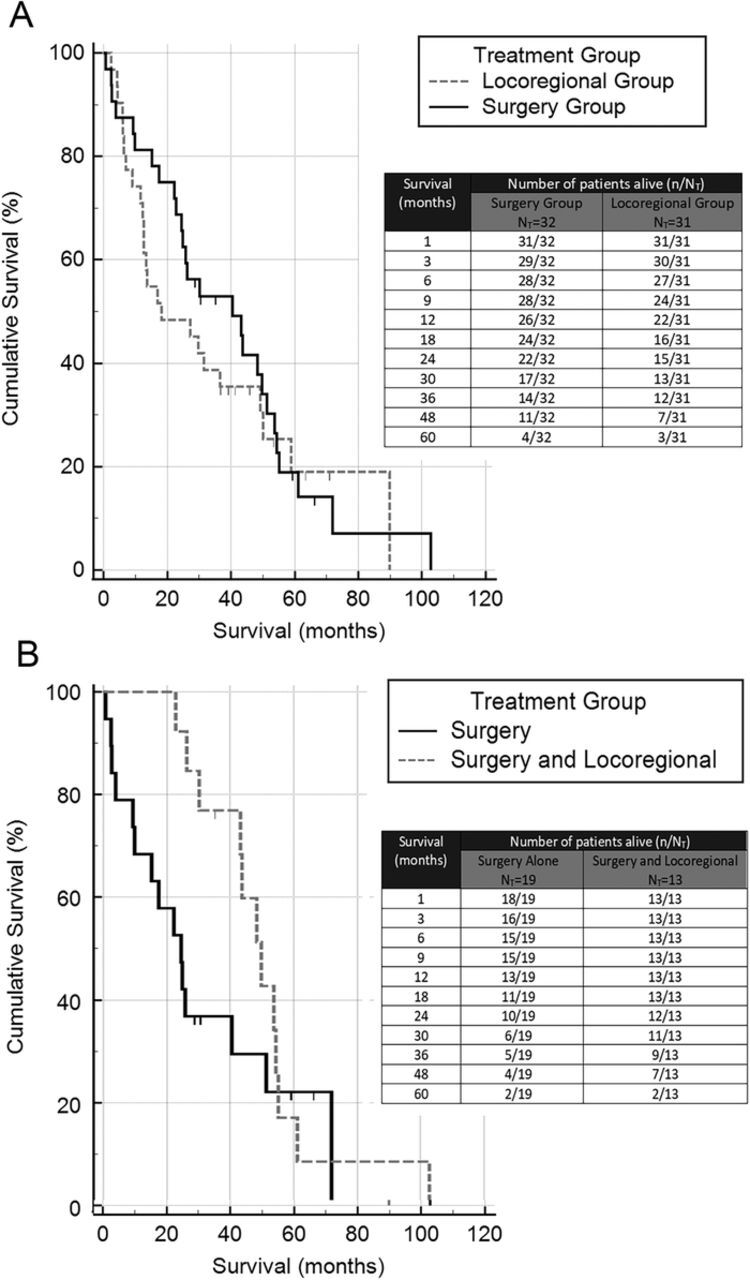
**Kaplan-Meier survival analysis of overall survival based on the type of treatment (A and B).** N_T,_ total number of patients in the group.

Figures 2 and 3 are Kaplan-Meier survival curves by disease stage and treatment group. In Figure 2, the pairwise over strata log-rank test showed statistical significance of each stage when compared to the others (*P*=0.001 to *P*=0.046), except between the early and intermediate stages of the disease (*P*=0.304). In Figure 3A, despite providing higher survival rates, the superiority of surgery did not reach statistical significance (log-rank test *P*=0.426). Figure 3B shows that patients who underwent surgery followed by locoregional treatment had significantly higher survival rates than patients who underwent surgery alone (log-rank test *P*=0.038).

## DISCUSSION

This retrospective cohort study, despite being small, yielded several findings. Regarding the course of treatment, we demonstrated how the cooperation of physicians among different specialties, through an established oncology board that performs regular follow-up of patients, can lead to an individualized course of treatment for each patient. It is essential to highlight that our multidisciplinary oncology board decided on a course of treatment for several patients that was different from the treatment advised by guidelines. Other teams have reported such discrepancies, described as treatment stage migration.^12,13^

Regarding survival based on the stage of the disease, survival rates between the early and the intermediate stage of the disease, based on BCLC stage, were not statistically different. EASL clinical guidelines for the management of HCC acknowledge the affinity between these 2 stages. Therefore, the guidelines recommend the use of clinical data, molecular classes, and biomarkers, besides tumor burden, CTP class, and performance status, to further facilitate the treatment allocation and the understanding of outcome data.^3^

Regarding survival among the different therapy groups, even though hepatic resection provided higher mean overall survival and higher survival rates when compared to locoregional treatments, its superiority did not reach statistical significance. These findings highlight the importance of locoregional treatments as a therapeutic alternative to hepatic resection, particularly when surgery is not feasible. In addition, treatment with locoregional therapies following surgery proved to prolong survival when compared with patients who underwent surgery alone.

Several other studies have highlighted the importance of locoregional treatments in the management of HCC. TACE, besides being an alternative for surgery, can be used as a neoadjuvant agent for downstaging a tumor^14^; as a bridge, along with RFA, for patients awaiting liver transplantation^15,16^; to manage a ruptured HCC^17^; and as an adjuvant therapy after resection.^18^ For lesions <2 cm, RFA has shown equal survival rates to surgery, while the surgical risk is avoided.^19^

Nevertheless, deciding between hepatic resection and nonsurgical treatments is controversial, especially in specific clinical settings. An example is performing hepatic resection when the liver has low functional reserves. While some studies recommend hepatic resection only for patients with CTP class A and report poor surgical outcomes when hepatectomies are performed in patients with high Model for End Stage Liver Disease scores,^20,21^ several teams perform hepatic resections in patients with limited functional reserves (CTP class B).^22,23^ In addition, while current guidelines for the treatment of HCC recommend TACE for the intermediate stage of the disease,^3,7^ several surgical teams perform hepatectomies at this stage^24-27^ and even recommend that BCLC stages be revised.^24,27^

Finally, various biomarkers, such as microRNAs, could be used to classify HCC at a molecular level.^28,29^ The integration of molecular classification in the staging of the disease would allow for more targeted interventions and would clarify which patients are most likely to benefit from each therapeutic choice in the armamentarium of treatments in the management of HCC.^30^

Our study has limitations. The small patient population, especially in terms of the different treatment combinations, limits the statistical power of our results. The retrospective nature of our study could have introduced information bias. Further, this study includes only patients that either presented in our department or were referred to our department for potential surgical treatment. As a result, patients who were referred by secondary hospitals or private doctors to transplantation or internal medicine departments are not included in this study.

## CONCLUSION

Our study demonstrates the importance of locoregional therapies in the management of HCC both as an alternative to hepatic resection and as a therapy for recurrence following hepatic resection. In addition, we highlight the importance of a multidisciplinary oncology board in tailoring an individualized course of treatment for each patient that may not correspond to the treatment recommended by the guidelines based on the stage of the disease.

## Notes

This article meets the Accreditation Council for Graduate Medical Education and the American Board of Medical Specialties Maintenance of Certification competencies for Patient Care and Medical Knowledge.
